# Supplementary material for: Photocatalytic Synthesis of Coumarin Derivatives Using Visible-Light-Responsive Strawberry Dye-Sensitized Titanium Dioxide Nanoparticles
Source: Nanomaterials (Basel). 2023 Nov 22;13(23):3001. doi: 10.3390/nano13233001 (PMC10707762; doi:10.3390/nano13233001)
Supplement: Supplementary file 1 [file nanomaterials-13-03001-s001.zip › nanomaterials-2632274-supplementary.pdf]

Supplementary materials

Photocatalytic Synthesis of Coumarin Derivatives Using Visible-Light-Responsive Strawberry Dye-Sensitized Titanium Dioxide Nanoparticles

Mshari A. Alotaibi [1,*], Abdulrahman I. Alharthi [1], Talal F. Qahtan [2], Satam Alotibi [2], Amani M. Alansi [3] and Md. Afroz Bakht [1]

[1] Chemistry Department, College of Science and Humanities, Prince Sattam Bin Abdulaziz University, P.O. Box 83, Al-Kharj 11942, Saudi Arabia; a.alharthi@psau.edu.sa (A.I.A.); m.bakht@psau.edu.sa (M.A.B.)

[2] Department of Physics, College of Science and Humanities, Prince Sattam Bin Abdulaziz University, P.O. Box 173, Al-Kharj 11942, Saudi Arabia; t.qahtan@psau.edu.sa (T.F.Q.); sf.alotibi@psau.edu.sa (S.A.)

[3] Chemistry Department, King Saud University, P.O. Box 2455, Riyadh 12372, Saudi Arabia; amanialansi92@gmail.com

* Correspondence: alosaimi@psau.edu.sa

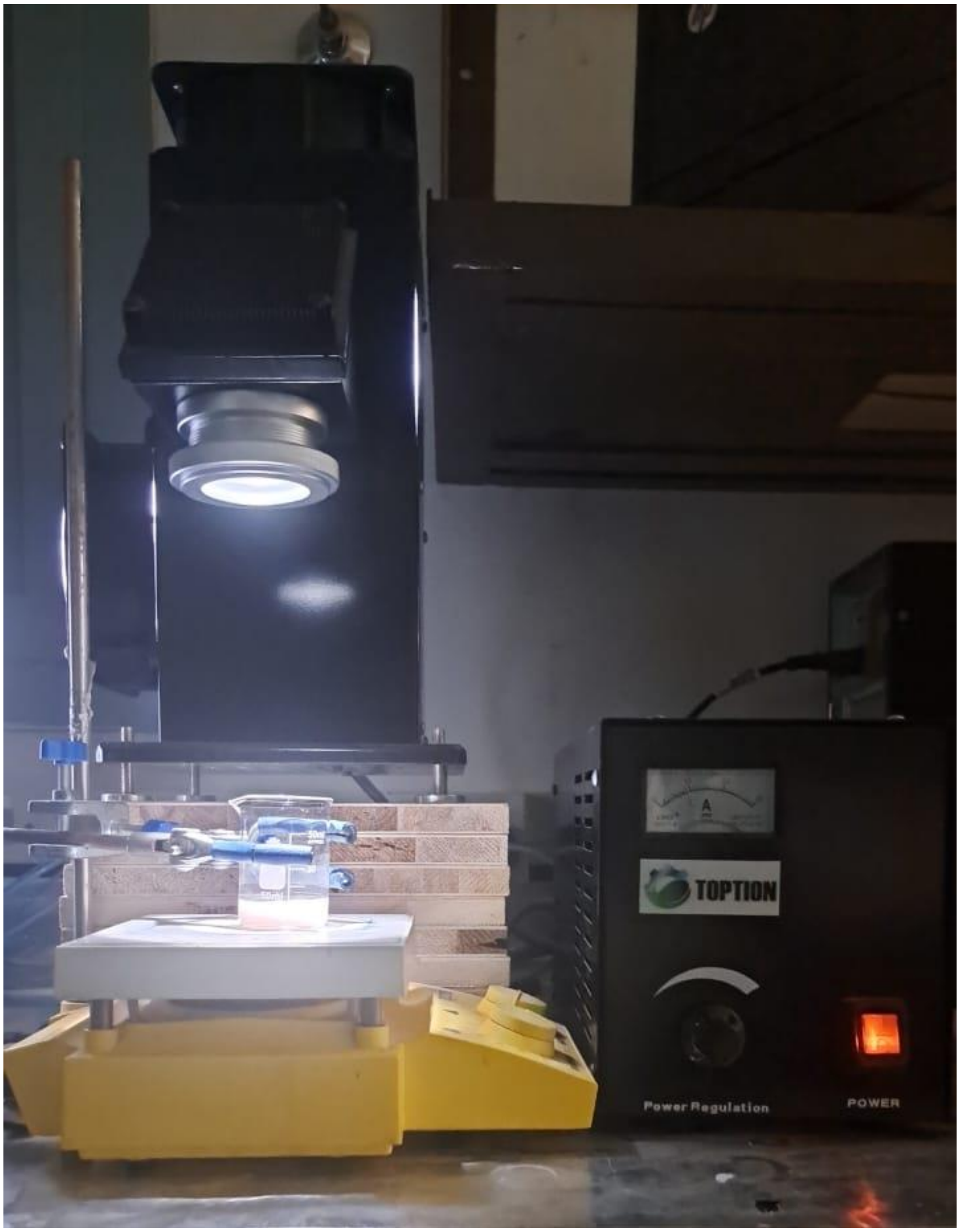

Figure S1. The digital images for reaction setup along with lamb sources (UV–vis light using a 500W Xenon/UV cut-off filter (< 420 nm)).

Figure S2. NMR Data of synthesized compounds **(4a–h).**

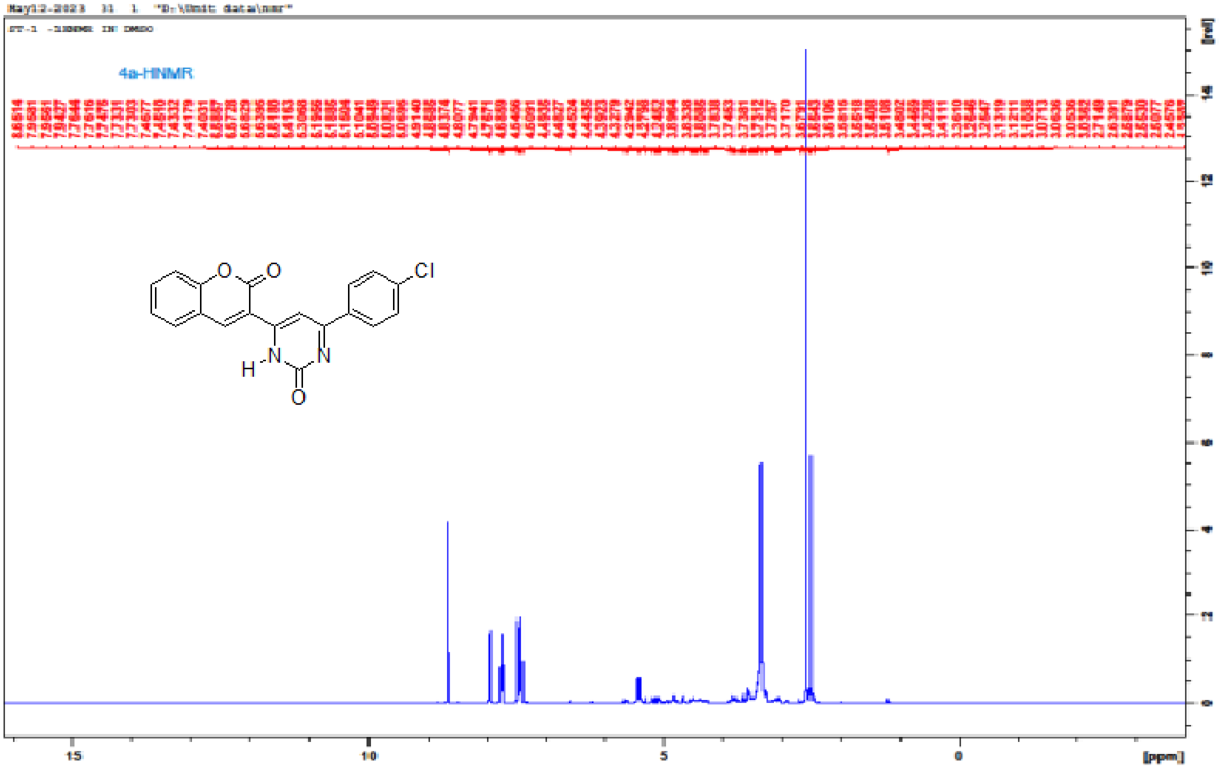

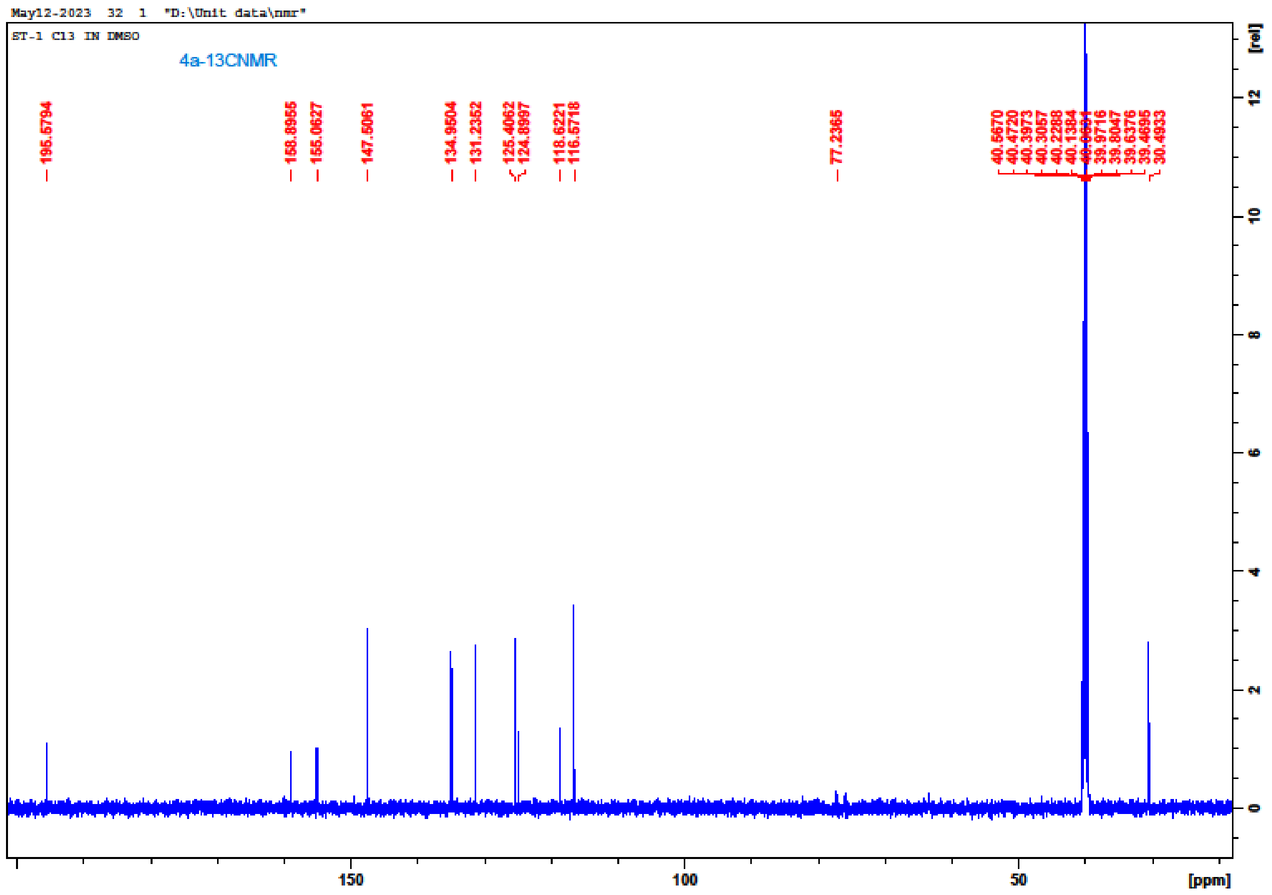
May12-2023 32 1 "D:\Unit data\nmr"
ST-1 C13 IN DMSO
4a-13CNMR
195.5794
158.8955
155.0627
147.5061
134.9504
131.2352
125.4062
124.8997
118.6221
116.5718
77.2365
40.5670
40.4720
40.3973
40.3057
40.2288
40.1384
39.9716
39.8047
39.6376
39.4695
30.4933
150
100
50
[ppm]
[rel]
12
10
8
6
4
2
0

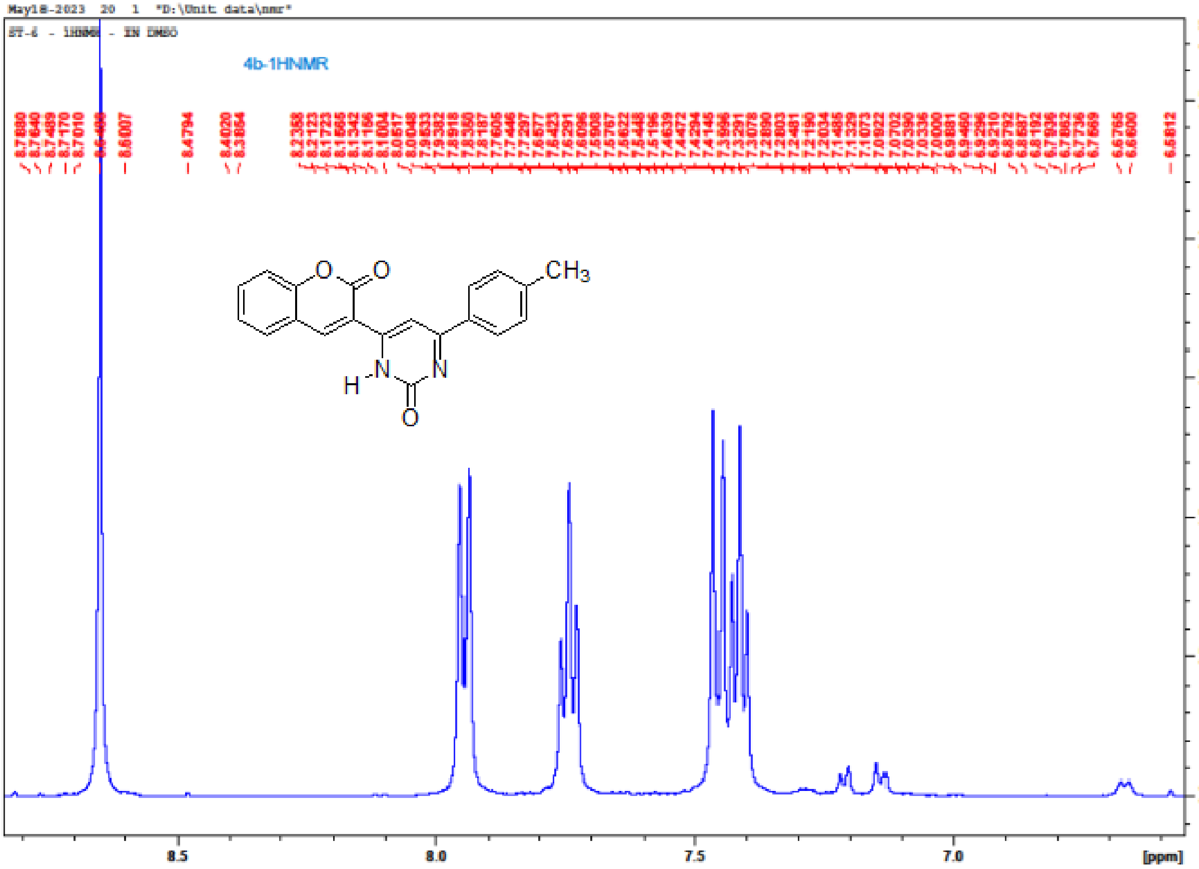

4b-1HNMR
CH3
O
O
N
H
N
O
[rel]
[ppm]
8.5
8.0
7.5
7.0

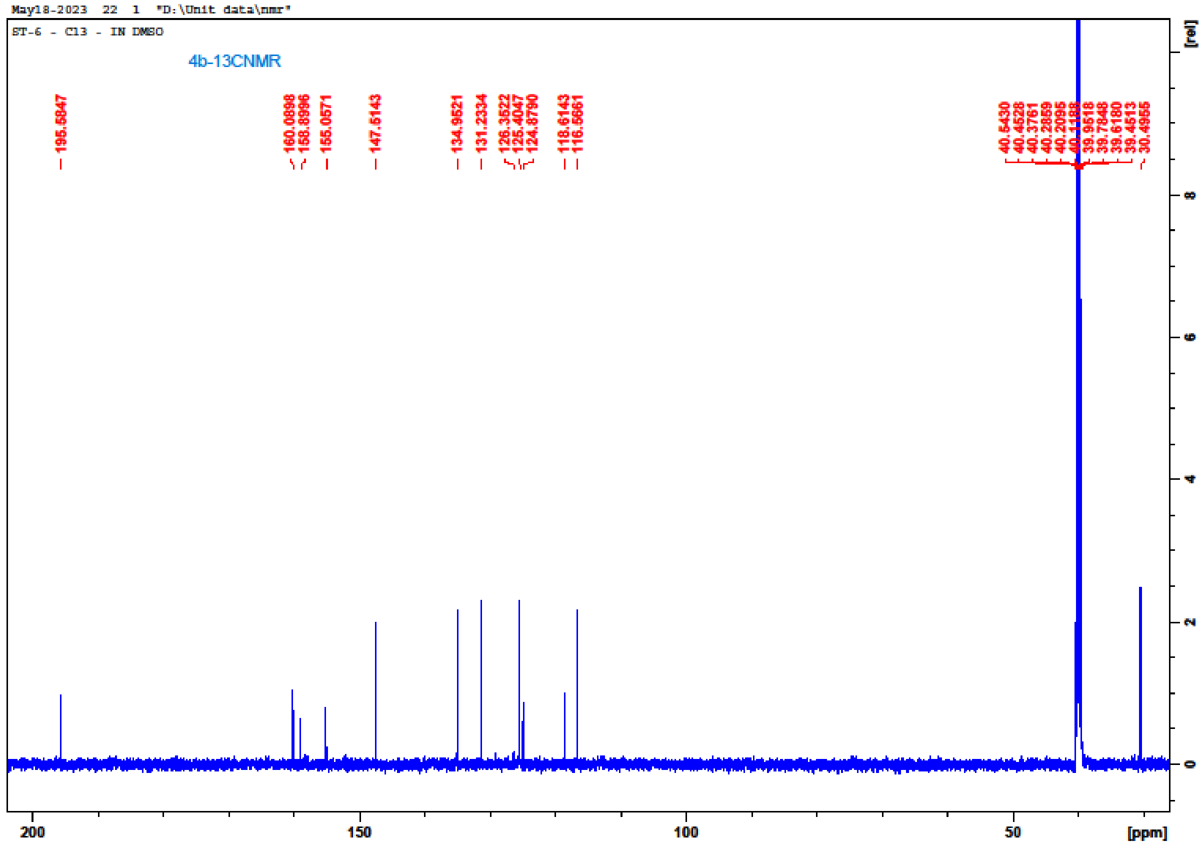
May18-2023 22 1 "D:\Unit data\nmr"
ST-6 - C13 - IN DMSO
4b-13CNMR
195.5847
160.0898
158.8996
155.0571
147.5143
134.9521
131.2334
126.3522
125.4047
124.8790
118.6143
116.5661
40.5430
40.4528
40.3761
40.2859
40.2095
40.1188
39.9518
39.7848
39.6180
39.4513
30.4955
[rel]
[ppm]

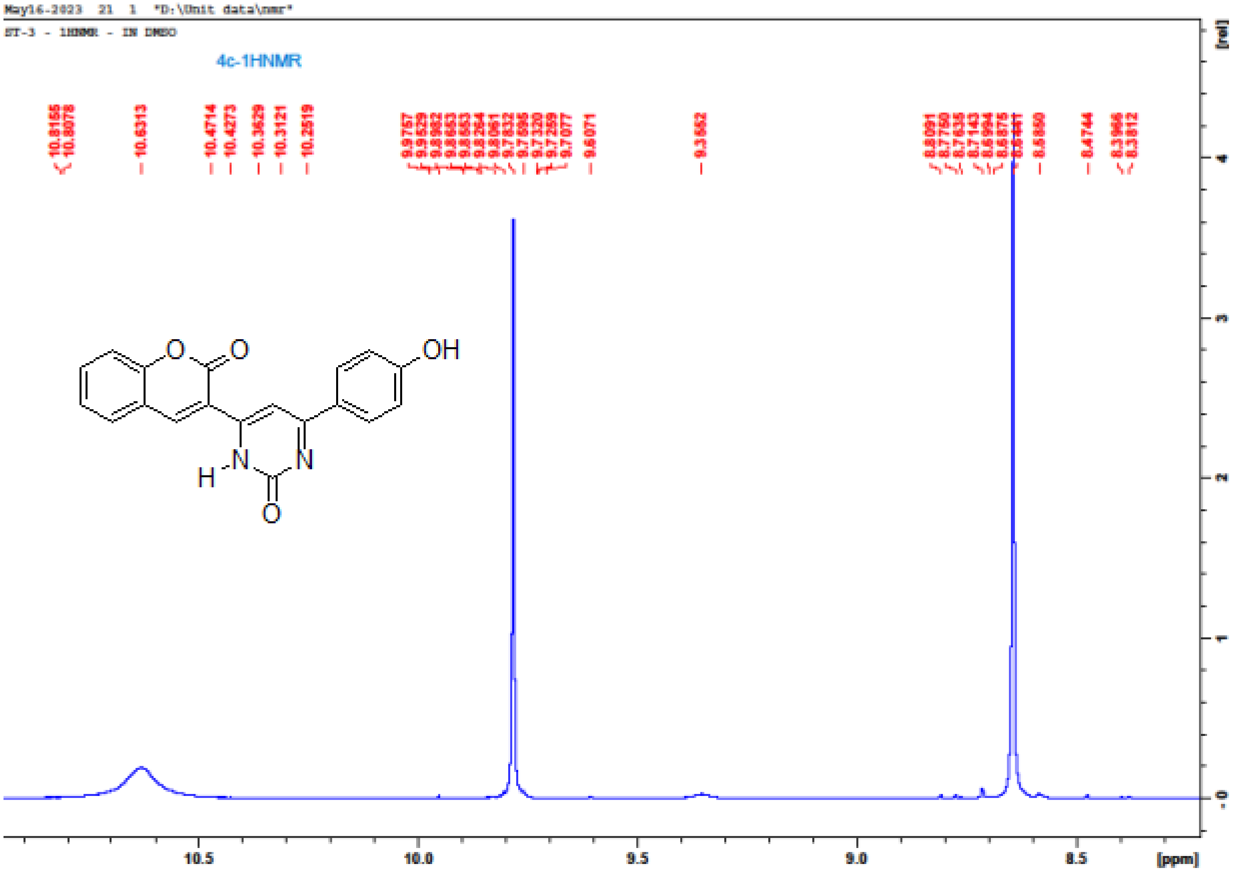
May16-2023 21 1 "D:\Unit data\nmr"
ST-3 - 1HNMR - IN DMSO
4c-1HNMR
OH
O
N
H
10.5
10.0
9.5
9.0
8.5
[ppm]
[rel]

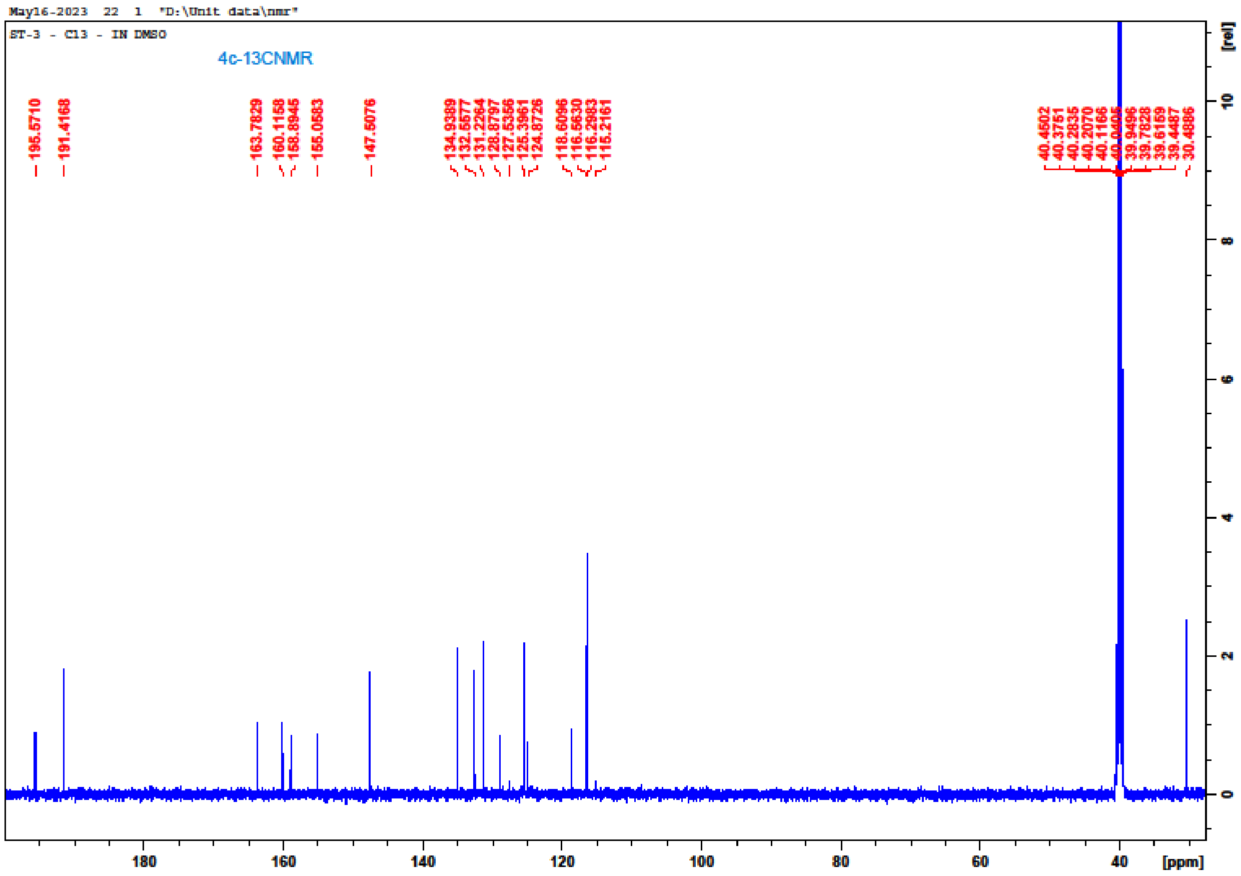

May16-2023 22 1 "D:\Unit data\nmr"
ST-3 - C13 - IN DMSO
4c-13CNMR
195.5710
191.4168
163.7829
160.1158
158.8945
155.0583
147.5076
134.9389
132.5577
131.2264
128.8797
127.5356
125.3961
124.8726
118.6096
116.5630
116.2983
115.2161
40.4502
40.3751
40.2835
40.2070
40.1166
40.0405
39.9496
39.7828
39.6159
39.4487
30.4886
180
160
140
120
100
80
60
40
[ppm]
10
8
6
4
2
0
[rel]

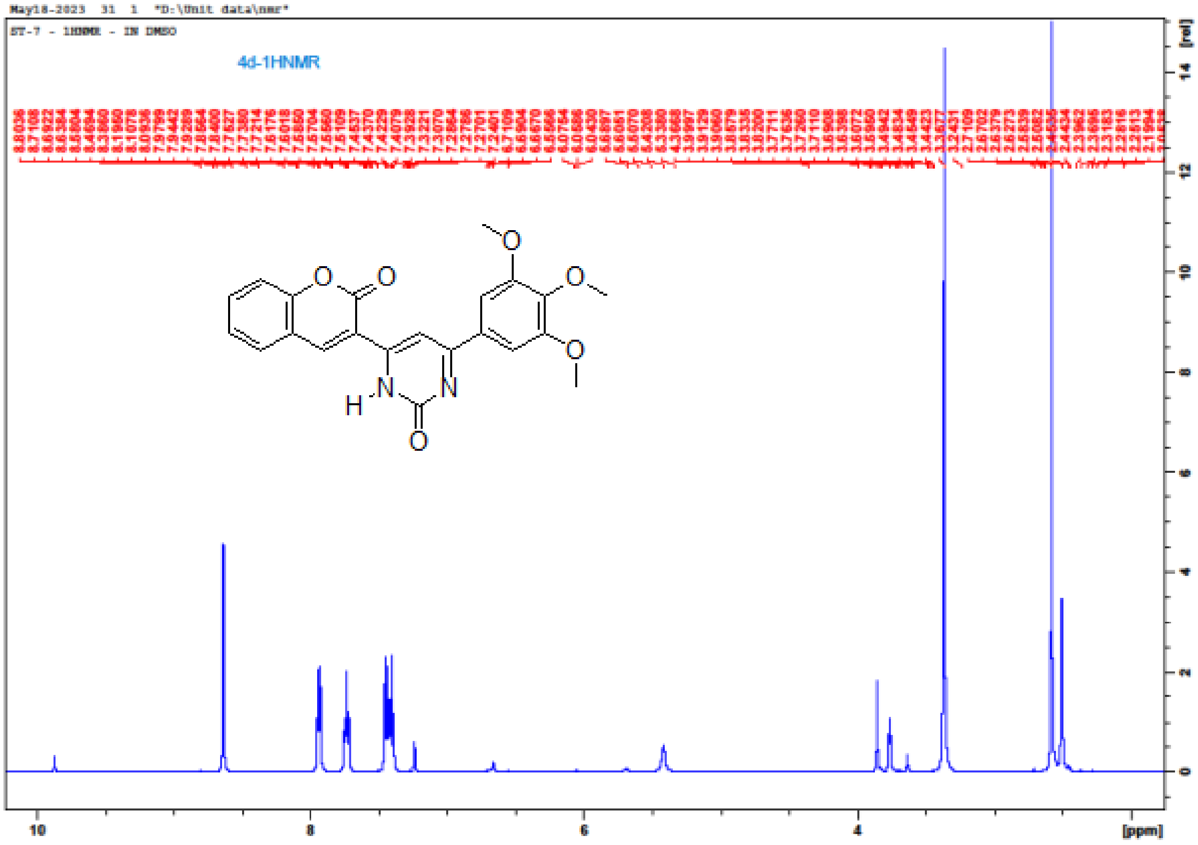
May18-2023 31 1 "D:\Unit data\nmr"
ST-7 - 1HNMR - IN DMSO
4d-1HNMR
[rel]
[ppm]

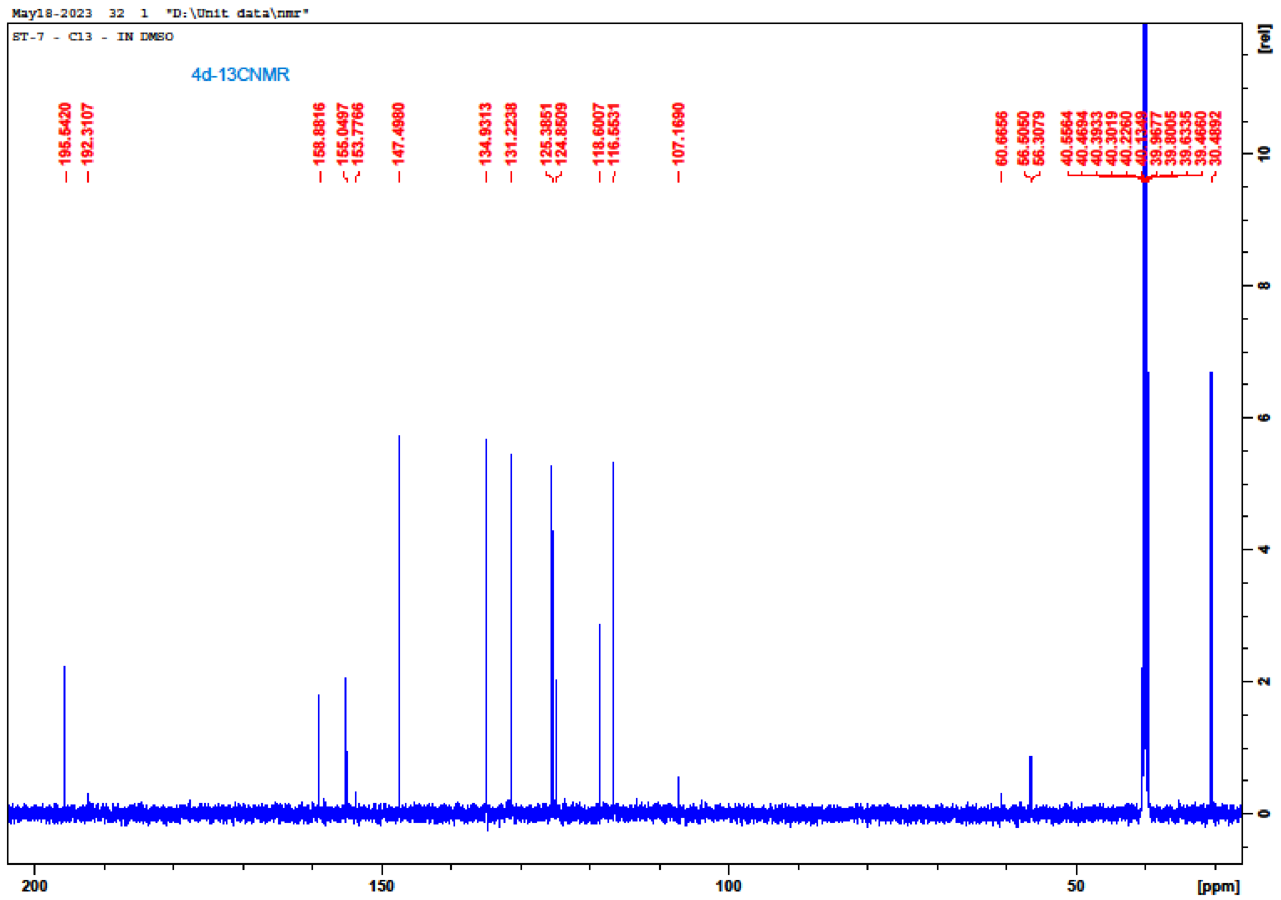
May18-2023 32 1 "D:\Unit data\nmr"
ST-7 - C13 - IN DMSO
4d-13CNMR
195.5420
192.3107
158.8816
155.0497
153.7766
147.4980
134.9313
131.2238
125.3851
124.8509
118.6007
116.5531
107.1690
60.6656
56.5050
56.3079
40.5564
40.4694
40.3933
40.3019
40.2260
40.1349
39.9677
39.8005
39.6335
39.4660
30.4892
[rel]
[ppm]

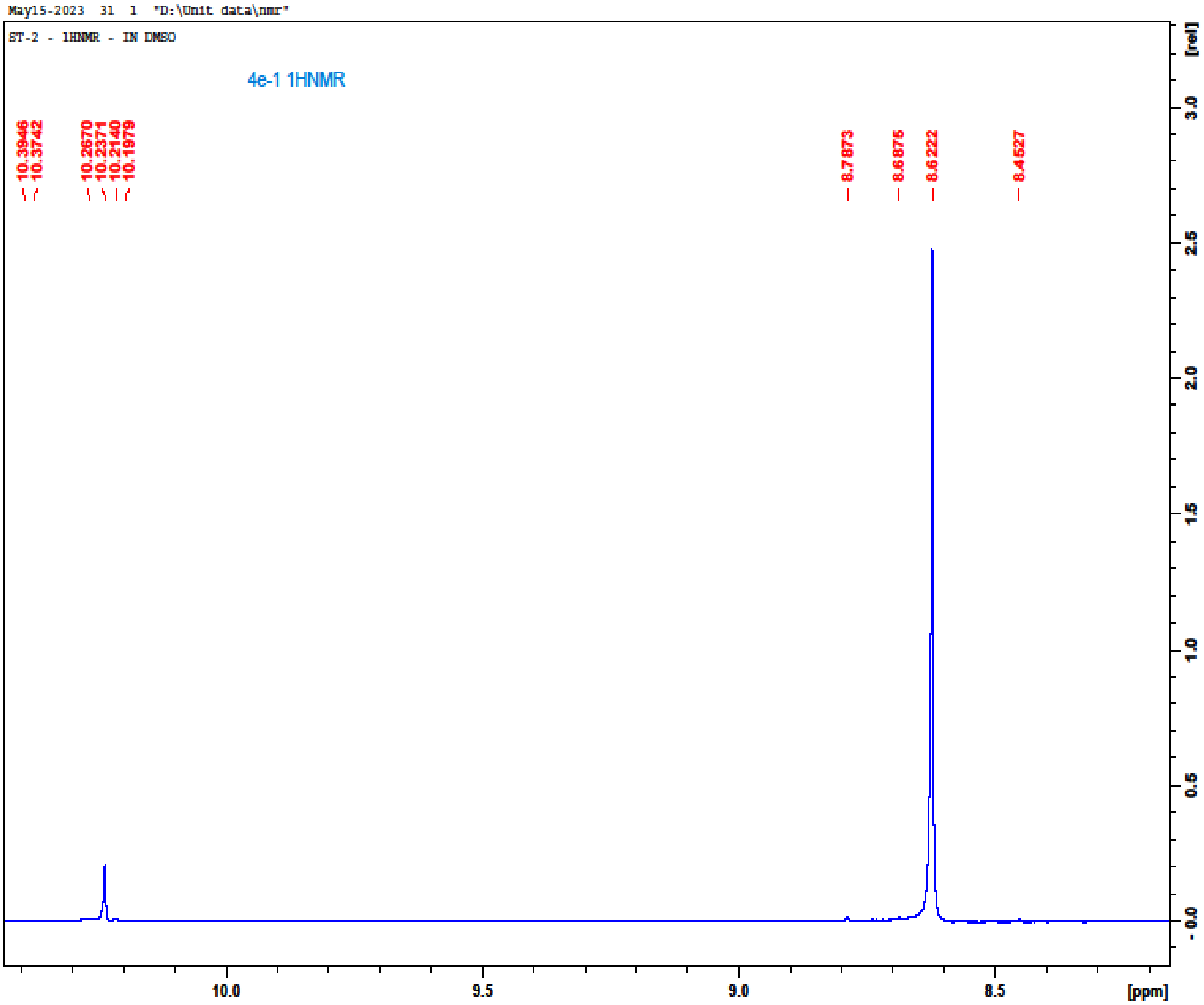
May15-2023 31 1 "D:\Unit data\nmr"
ST-2 - 1HNMR - IN DMSO
4e-1 1HNMR
10.3946
10.3742
10.2670
10.2371
10.2140
10.1979
8.7873
8.6875
8.6222
8.4527
[rel]
3.0
2.5
2.0
1.5
1.0
0.5
0.0
10.0
9.5
9.0
8.5
[ppm]

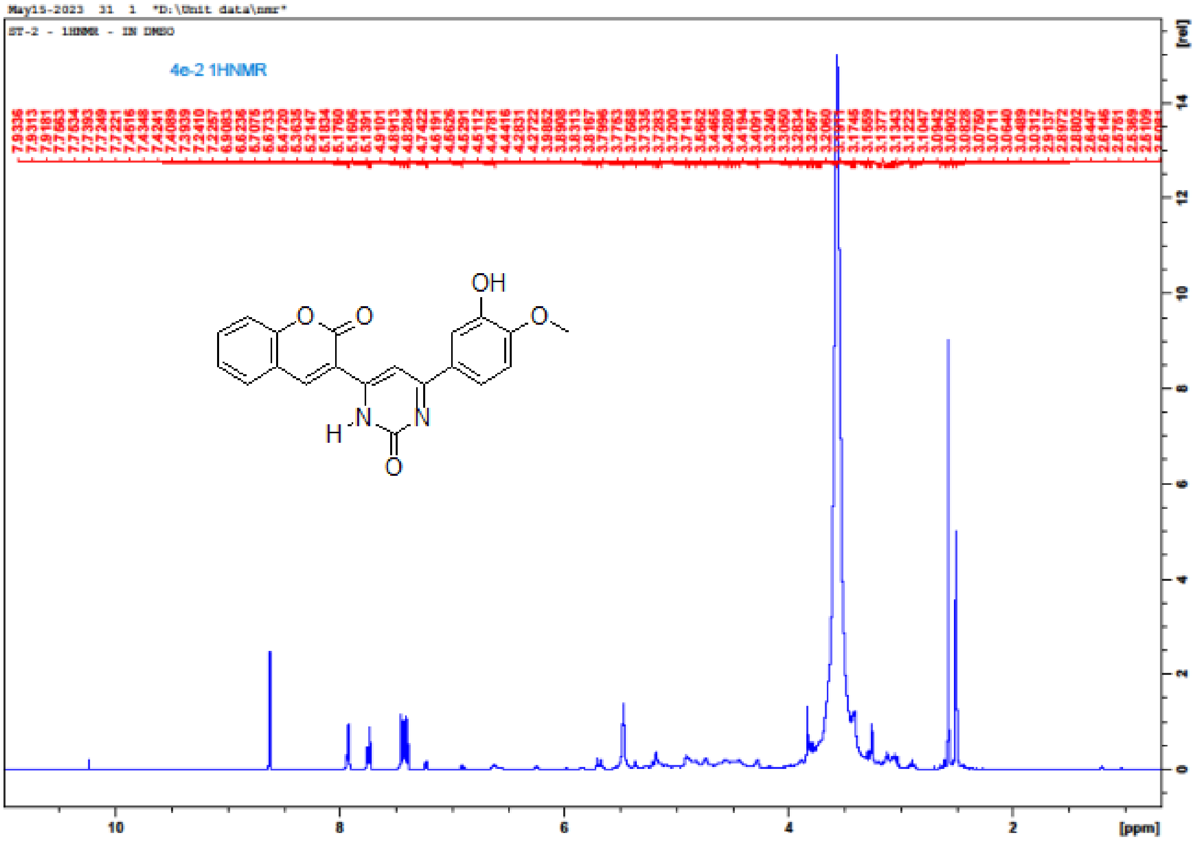

May15-2023 31 1 "D:\Unit data\nmr"
ST-2 - 1HNMR - IN DMSO
4e-2 1HNMR
OH
O
H
N
[rel]
14
12
10
8
6
4
2
0
10
8
6
4
2
[ppm]

May15-2023 32 1 "D:\Unit data\nmr"

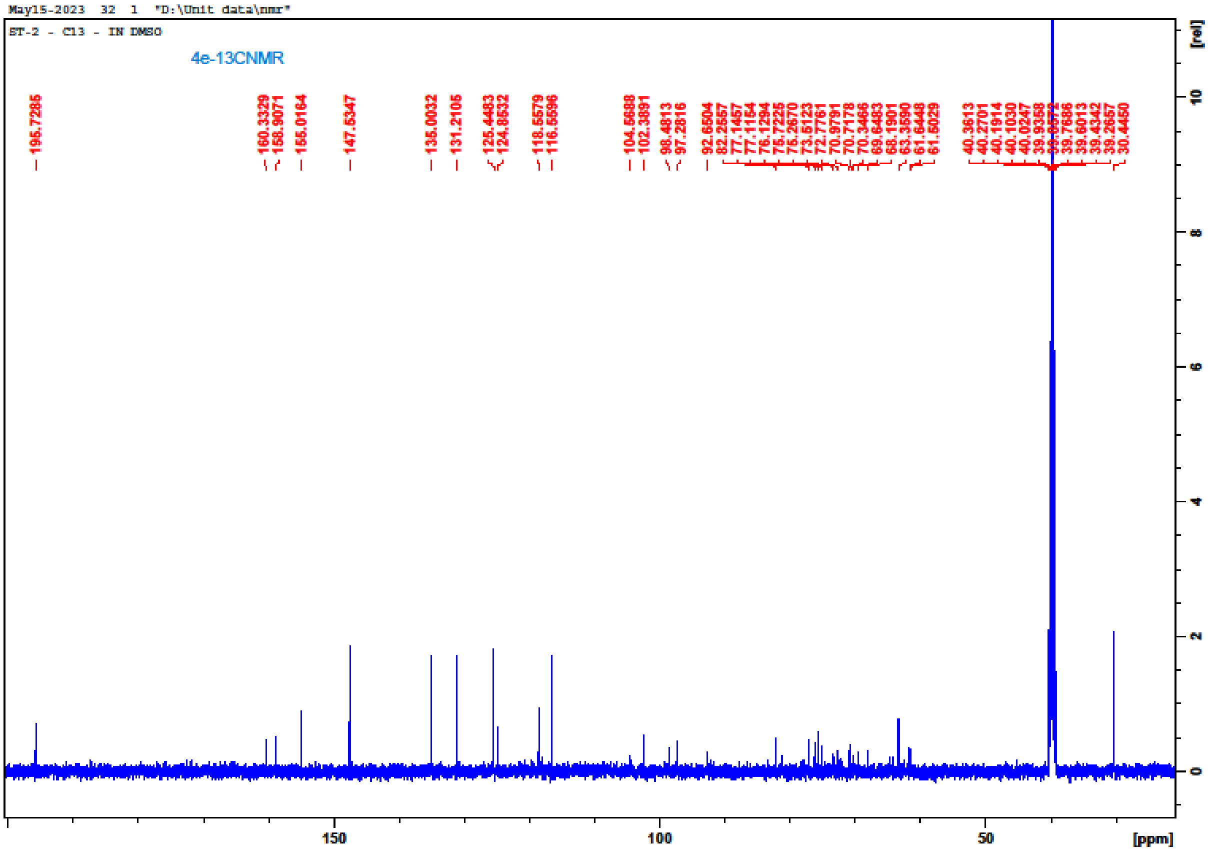

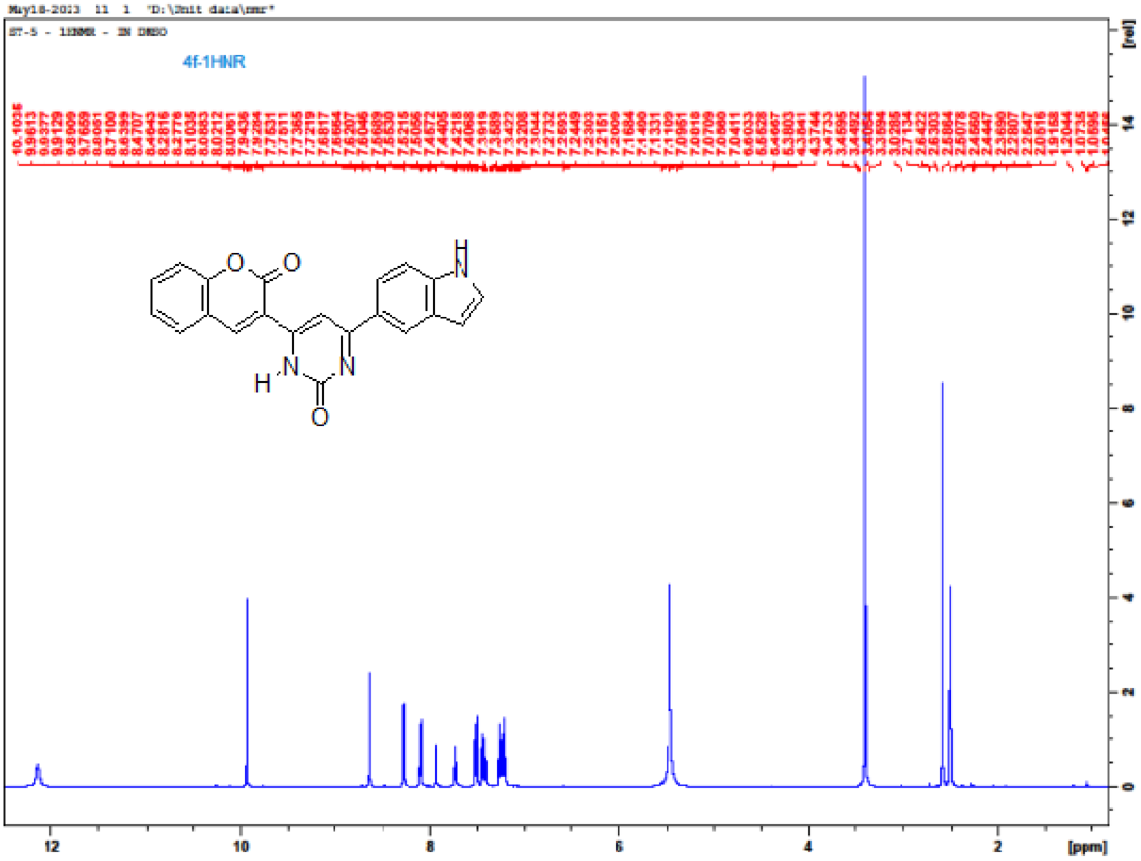
4f-1HNR
[rel]
[ppm]

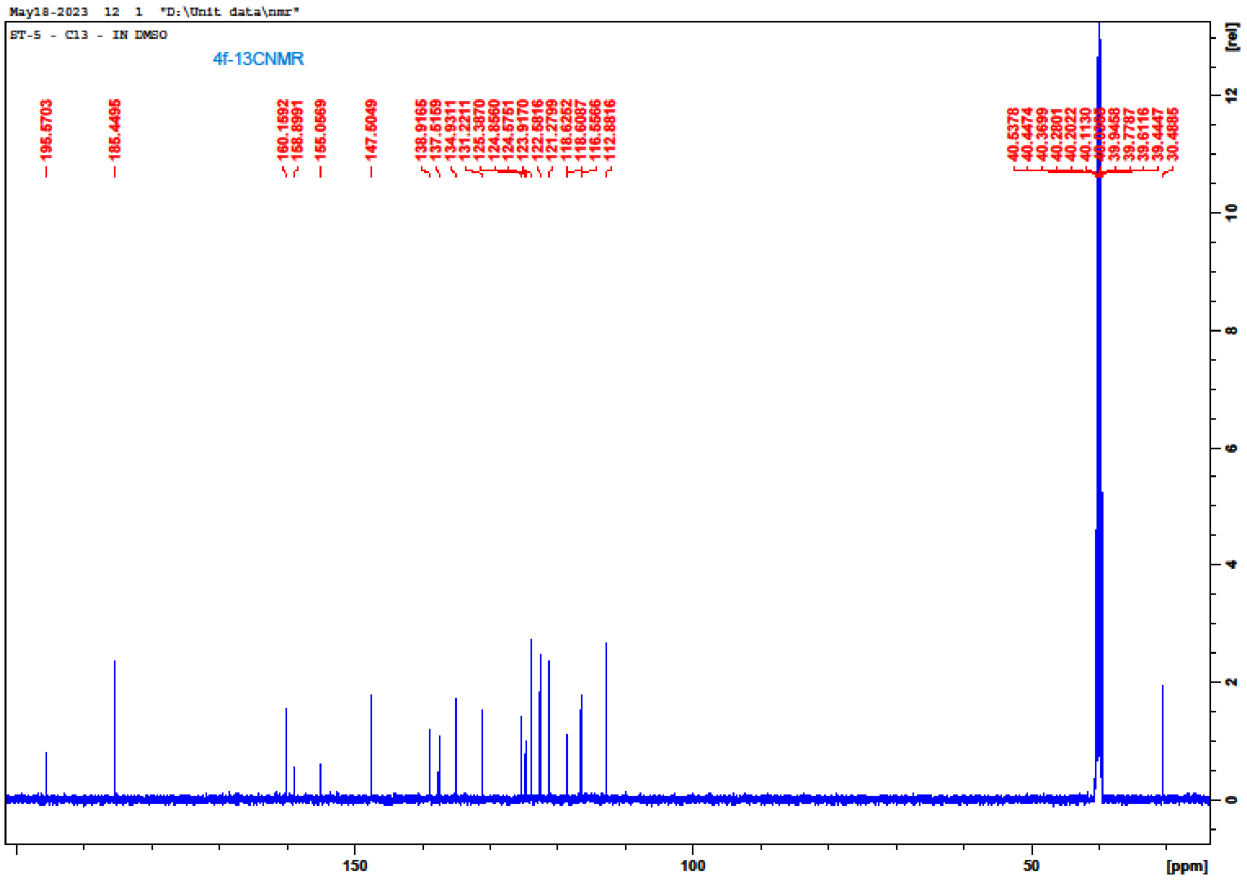

May18-2023 12 1 "D:\Unit data\nmr"
ST-5 - C13 - IN DMSO
4f-13CNMR
195.5703
185.4495
160.1592
158.8991
155.0569
147.5049
138.9165
137.5159
134.9311
131.2211
125.3870
124.8560
124.5751
123.9170
122.5816
121.2799
118.6252
118.6087
116.5566
112.8816
40.5378
40.4474
40.3699
40.2801
40.2022
40.1130
39.9458
39.7787
39.6116
39.4447
30.4885
150
100
50
[ppm]
12
10
8
6
4
2
0
[rel]

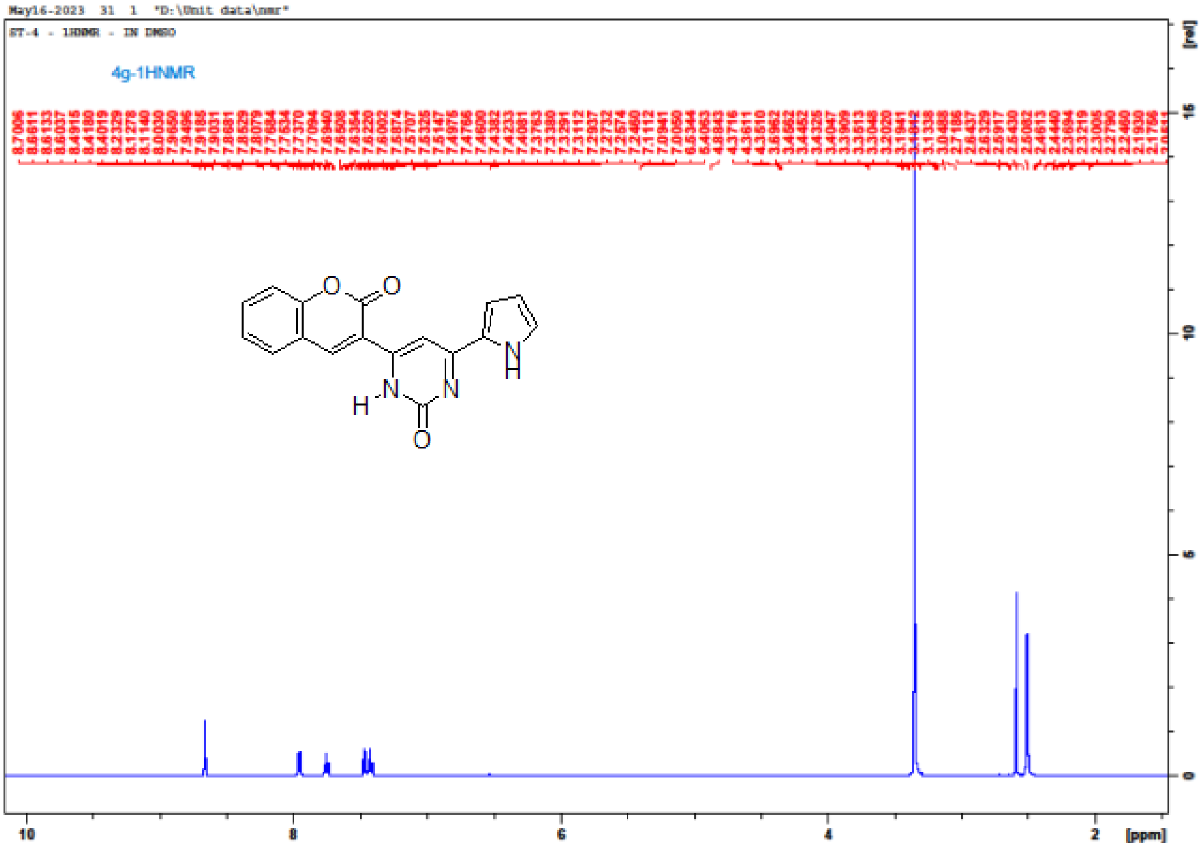
May16-2023 31 1 "D:\Unit data\nmr"
ST-4 - 1HNMR - IN DMSO
4g-1HNMR
[rel]
[ppm]

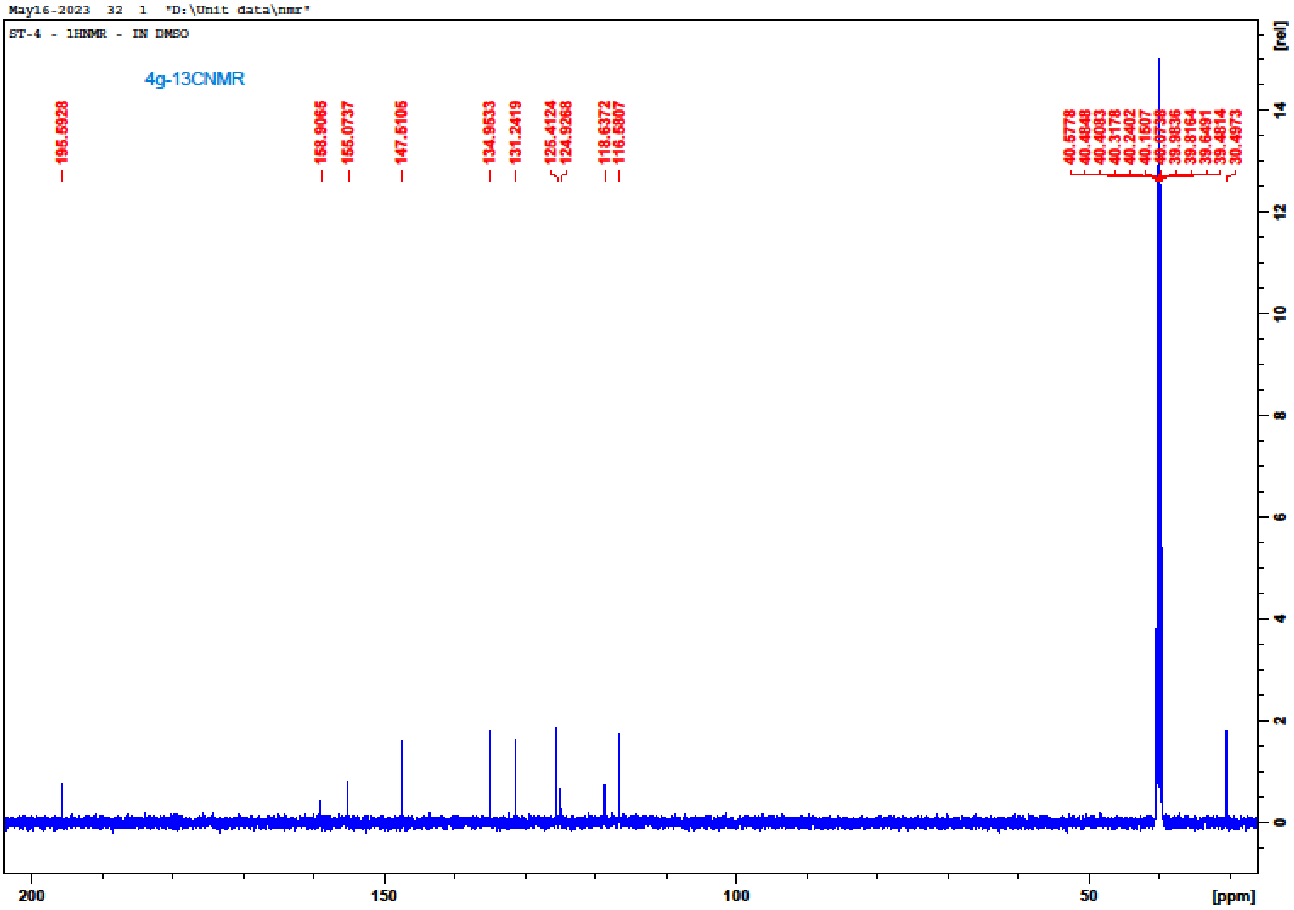
May16-2023 32 1 "D:\Unit data\nmr"
ST-4 - 1HNMR - IN DMSO
4g-13CNMR
195.5928
158.9065
155.0737
147.5105
134.9533
131.2419
125.4124
124.9268
118.6372
116.5807
40.5778
40.4848
40.4083
40.3178
40.2402
40.1507
39.9836
39.8164
39.6491
39.4814
30.4973
[rel]
14
12
10
8
6
4
2
0
200
150
100
50
[ppm]

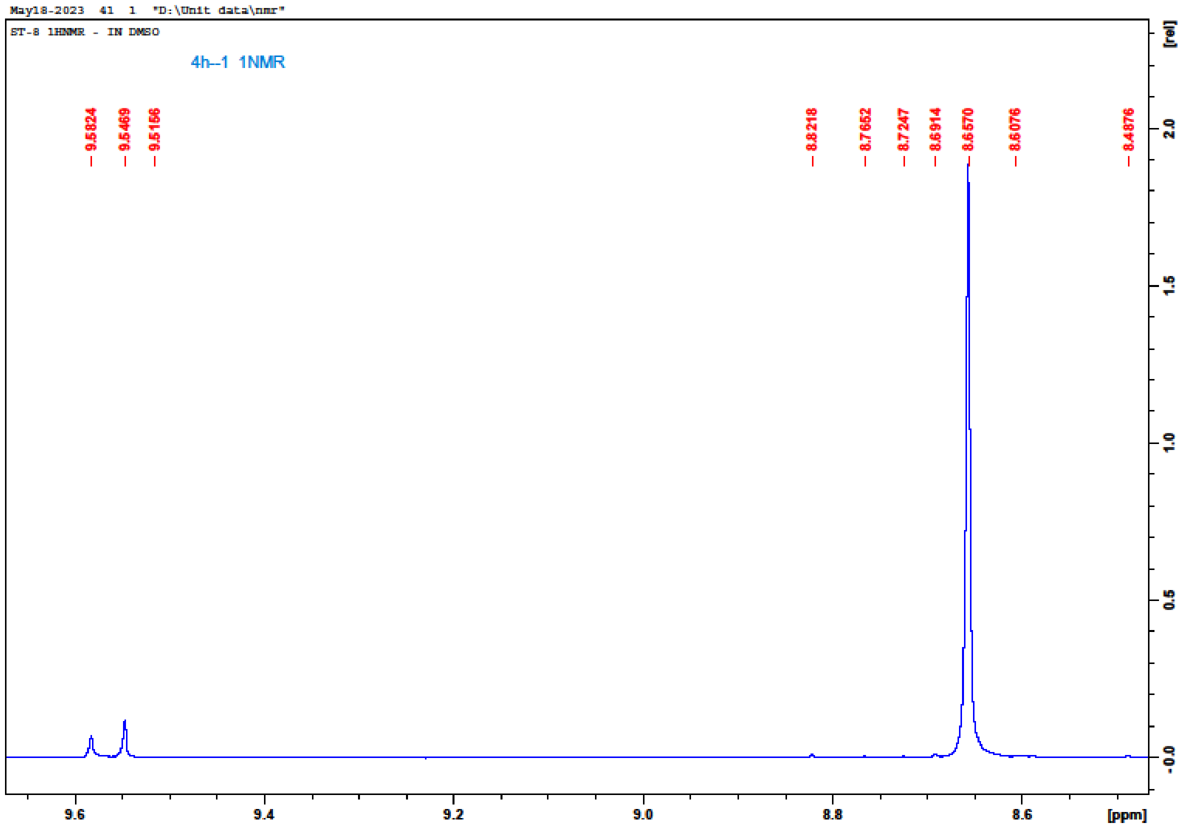

May18-2023 41 1 "D:\Unit data\nmr"
ST-8 1HNMR - IN DMSO
4h--1 1NMR
9.5824
9.5469
9.5156
8.8218
8.7652
8.7247
8.6914
8.6570
8.6076
8.4876
[rel]
2.0
1.5
1.0
0.5
-0.0
9.6
9.4
9.2
9.0
8.8
8.6
[ppm]

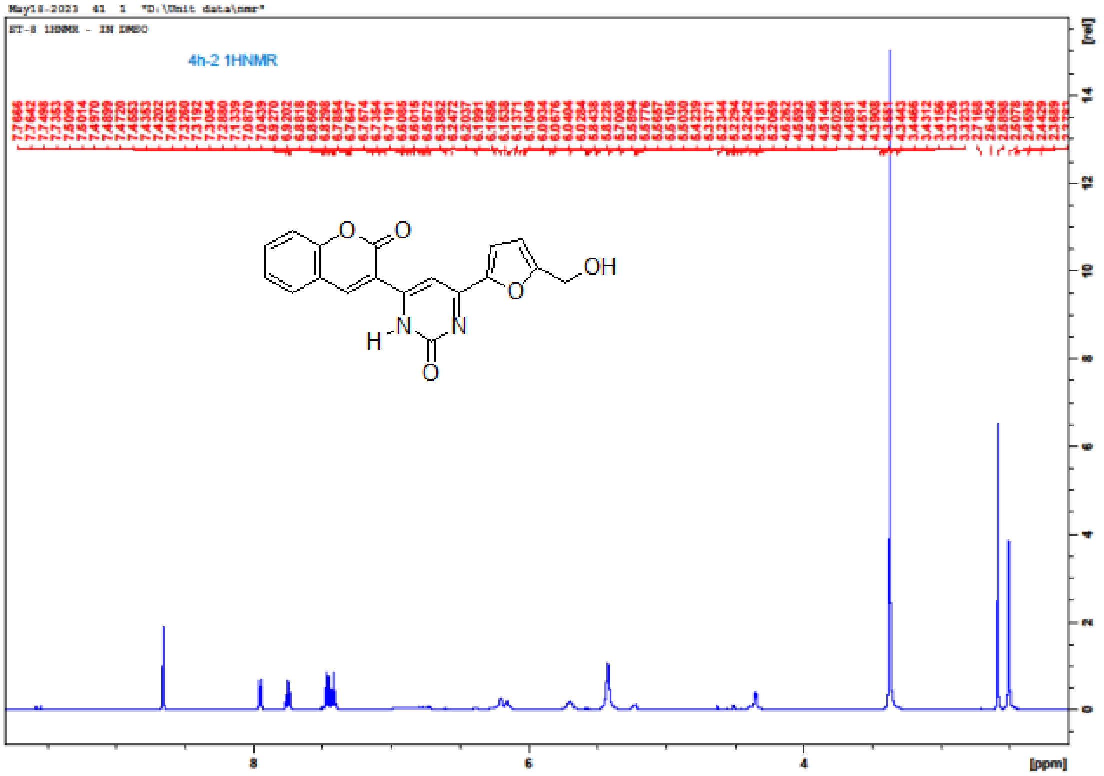
May18-2023 41 1 "D:\Unit data\nmr"
ST-8 1HNMR - IN DMSO
4h-2 1HNMR
[rel]
[ppm]
OH
H
N
O

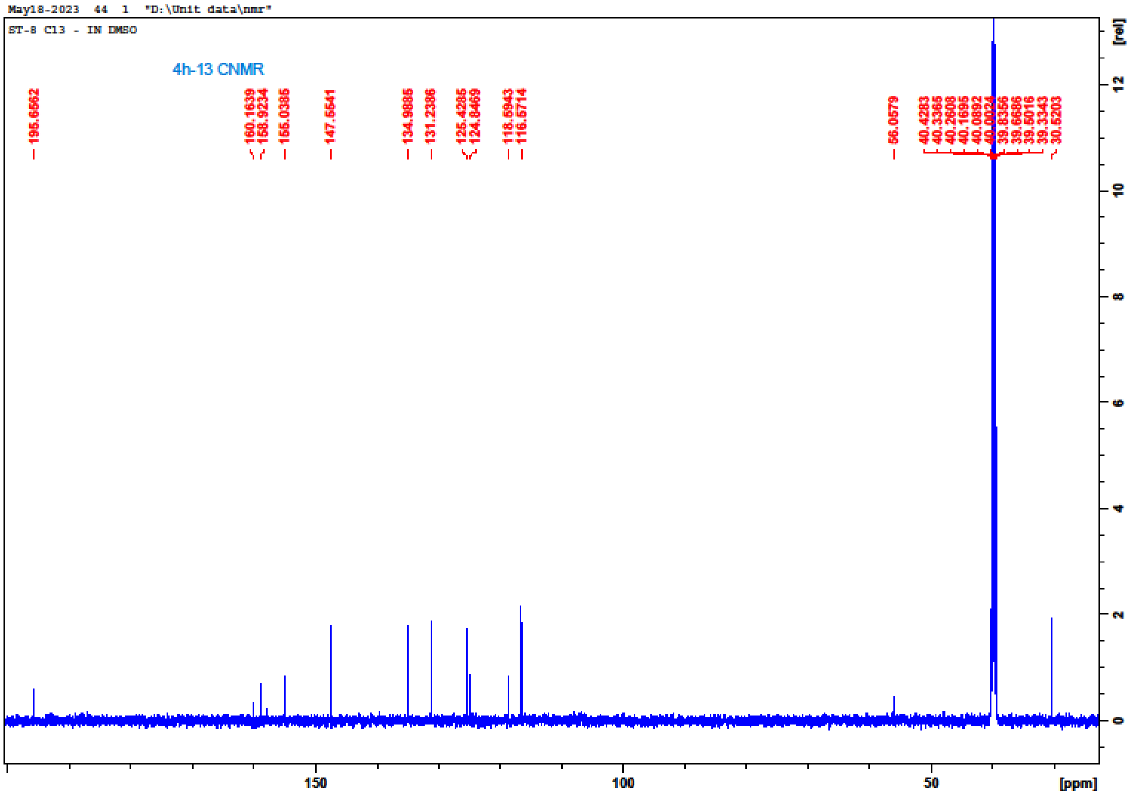
May18-2023 44 1 "D:\Unit data\nmr"
ST-8 C13 - IN DMSO
4h-13 CNMR
195.6562
160.1639
158.9234
155.0385
147.5541
134.9885
131.2386
125.4285
124.8469
118.5943
116.5714
56.0579
40.4283
40.3365
40.2608
40.1695
40.0892
40.0024
39.8356
39.6686
39.5016
39.3343
30.5203
150
100
50
[ppm]
[rel]
12
10
8
6
4
2
0
